# Orthohantavirus rodent hosts and genotypes in Southern South America: A narrative review

**DOI:** 10.1371/journal.pntd.0013489

**Published:** 2025-09-09

**Authors:** Natalia Ortiz, Juan Diego Pinotti, Verónica Andreo, Raúl Enrique González-Ittig, Cristina Noemí Gardenal

**Affiliations:** 1 Instituto de Diversidad y Ecología Animal (IDEA), CONICET and Universidad Nacional de Córdoba, Córdoba, Córdoba, Argentina; 2 Instituto de Altos Estudios Espaciales Mario Gulich (CONAE-UNC), Falda del Cañete, Córdoba, Argentina; 3 Consejo Nacional de Investigaciones Científicas y Técnicas (CONICET), Ciudad Autónoma de Buenos Aires, Buenos Aires, Argentina; 4 Cátedra de Genética de Poblaciones y Evolución, Facultad de Ciencias Exactas Físicas y Naturales, UNC, Córdoba, Córdoba, Argentina; Medizinische Universitat Wien, AUSTRIA

## Abstract

Orthohantaviruses, family Hantaviridae, are zoonotic agents that pose a significant public health threat, particularly in South America, where they cause severe respiratory illnesses in humans. Despite their importance, knowledge gaps remain regarding the distributions of both the viruses and their rodent hosts in Southern South America, a region characterized by a great complexity of viral genotypes and reservoirs. This review provides an updated overview of orthohantavirus hosts and their associated viral genotypes in Argentina, Chile, Paraguay, and Uruguay. Through an extensive literature review, we identified 14 rodent species that serve as reservoir hosts for 15 distinct orthohantavirus genotypes. These rodent hosts inhabit a variety of ecosystems, from forests and arid zones to grasslands and wetlands, and even modified or anthropized habitats, demonstrating a wide geographic and ecological range. Our findings highlight the diversity of orthohantaviruses in this region, reflecting their complex evolutionary histories. Maintaining an up-to-date knowledge base on this topic is essential for effective decision-making in public health.

## Introduction

In recent decades, there has been growing scientific interest in zoonoses, diseases transmitted from animals to humans. Most of these diseases are caused by wildlife-derived pathogens and can lead to a wide spectrum of illnesses in humans and animals, ranging from mild symptoms to severe and even fatal cases [1]. An example of such pathogens is the genus *Orthohantavirus*, rodent borne RNA viruses belonging to the family Hantaviridae, that can cause severe respiratory and hemorrhagic diseases in humans. The main disease burden associated with orthohantaviruses are hemorrhagic fever with renal syndrome (HFRS) in the Old World and hantavirus cardiopulmonary syndrome or hantavirus pulmonary syndrome (HPS) in the New World [2]. HFRS is characterized by renal failure and hemorrhagic manifestations that vary from petechiae to severe internal bleeding [2]. HPS, on the other hand, is characterized by a sudden onset of symptoms, including fever, myalgia, and gastrointestinal distress, which rapidly progress to acute pulmonary edema, respiratory failure, and cardiogenic shock. HPS has a high fatality rate ranging from 12% to 40% in South America (SA), while HFRS has a considerably lower mortality rate (5%–10%). The absence of approved specific treatments for either disease amplifies their public health impact [3–5].

South America is a hotspot for orthohantavirus infections, with several genotypes identified and the highest rodent diversity in the world [6–9]. In this region, orthohantaviruses that cause human diseases are associated with native rodents of the subfamilies Sigmodontinae and Neotominae. Argentina and Brazil report the highest number of confirmed HPS cases in the continent and harbors a remarkable diversity of both orthohantaviruses and rodent species involved in their transmission [7,10,11]. Despite decades of research on reservoir hosts and their distributions in SA, which has led to the identification of new orthohantaviruses and host species [2,10], information on viral hosts in wildlife and their geographical ranges remains fragmented and insufficient [11].

Orthohantavirus risk is typically estimated by integrating data on rodent hosts—the most critical predictor—along with climatic, environmental, and land-use factors, human behavior, and clinical indicators [12,13]. Usually, the initial steps to estimate risk include identifying the reservoir species, delineating its geographic range, determining the extent of pathogen circulation, and assessing the relative risk to humans [14]. According to the most widely accepted conceptual framework, a reservoir host is defined as a species in which a pathogen is stably maintained over time within its populations [15,16]. Spillover hosts, on the other hand, are individuals or species that may become infected but do not support sustained transmission cycles. While they can contribute to pathogen persistence during periods of low prevalence, their role in transmission is generally limited and depends on specific ecological and epidemiological conditions [2]. Because the distribution of a viral strain is inherently constrained by that of its reservoir host, studying orthohantavirus host species is key to understanding transmission dynamics and developing targeted surveillance and control strategies [11].

The diversity of the genus *Orthohantavirus* in SA has been partially elucidated through various approaches. While phylogenetic analyses have identified novel orthohantavirus lineages in rodent species [7,17], field surveys have also detected antibodies in non-rodent mammals, like soricomorphs (moles and shrews) [18]. These findings indicate that viral hosts in SA may be more diverse than previously thought. However, significant knowledge gaps remain, particularly due to the challenge of detecting and characterizing viral lineages when different host species may carry multiple orthohantavirus genotypes [19,20].

A decade ago, a seminal review of rodent-associated orthohantaviruses in Southern South America (SSA), encompassing Argentina, Chile, Paraguay, and Uruguay, was published [21]. Concurrently, a comprehensive synthesis of orthohantavirus ecology in Brazil was carried out [10]. Since then, the region has seen the emergence of new orthohantavirus genotypes, the identification of novel rodent reservoir hosts, and the reclassification of some species previously considered reservoirs, which are now recognized as spillover hosts [22–24]. In addition, growing evidence highlights the influence of environmental and anthropogenic factors on virus dynamics and host distribution [10,21,25,26]. However, an updated regional perspective for SSA is lacking. This review aims to fill that gap by providing an updated synthesis of rodent hosts and their associated orthohantaviruses in SSA, thereby complementing existing studies and contributing to a more integrated understanding of orthohantavirus ecology on the continent. Understanding their diversity and distribution is essential for designing effective public health strategies, improving surveillance, and guiding interventions for better management and prevention of orthohantavirus diseases in SA.

## Methods

To perform this narrative review, we carried out a bibliographic search of scientific articles in the web engines PubMed, Web of Science, and Google Scholar. We searched for articles containing any combination of the following keywords in the title or abstract: “orthohantavirus”, “reservoir”, “host”, “South America”, “rodents”, “Argentina”, “Chile”, “Paraguay,” and “Uruguay”. No specific time frame was applied in the search. We aimed to compile all available evidence regardless of publication date or language, with special attention to recent updates in taxonomy and host-virus associations. Rodent species were included in this review if they were associated with any orthohantavirus strain—regardless of pathogenicity—and if their distribution encompassed, at least in part, Argentina, Chile, Paraguay, and/or Uruguay. Rodent species were considered as a reservoir host if there was molecular confirmation of viral replication and shedding, and if they were documented as hosts in at least two independent studies. Species in which only viral or antibody presence was detected are discussed in the section Spillover hosts.

Host’s taxonomic classification and distributions were mainly extracted from [9] and updated when newer publications were available (*e.g.*, the distribution of *Calomys laucha* considers the observations of [27]). All orthohantavirus genotypes identified by the reporting articles were included in the review even if they are not yet classified in any official *Orthohantavirus* species by the ICTV (see section Genotype diversity in SSA for more details).

## Results and discussion

### Reservoir hosts

**Fig 1:**
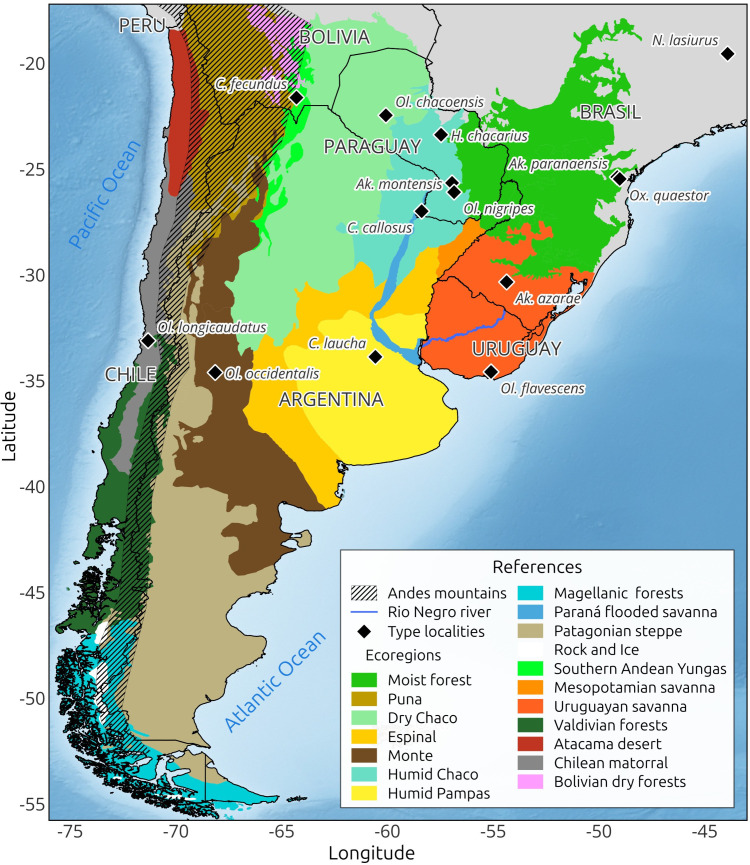
Type localities of orthohantavirus reservoir hosts detected in Southern South America. Ecoregions and geographic features are represented by colors/patterns. The map was created with QGIS 3.40.3-Bratislava (www.qgis.org). Base map and administrative borders were downloaded from Natural Earth (https://www.naturalearthdata.com/downloads/) and ecoregions from the World Wildlife Fund site (https://www.worldwildlife.org/publications/terrestrial-ecoregions-of-the-world) [80]. The Andes Mountains were delimited using the digital elevation model ASTGTM v003 from the U.S. Geological Survey (https://lpdaac.usgs.gov/products/astgtmv003/).

A total of 108 studies were revised, 47 documenting the association between rodent species and viral genotypes, and 61 describing host’s distributions. Our findings indicate that, to date, there are 14 rodent species recognized as reservoir hosts in SSA. This list includes species of the following genera: *Akodon* (2 species), *Calomys* (3 spp.), *Holochilus* (1 sp.), *Necromys* (1 sp.), *Oligoryzomys* (5 spp.), *Oxymycterus* (1 sp.), and *Rattus* (1 sp.). The host species identified, the viral genotype, and the known geographic distribution are presented in Table 1. The type localities of the reservoir host species, which refer to the places where these species were originally described, are depicted in Fig 1. These localities are shown along with ecoregions, administrative boundaries, and geographic features to facilitate the description of their distributions.

**Table 1:** Orthohantavirus reservoir host species, geographic distribution, orthohantavirus genotype hosted and their association with human disease.

Reservoir host	Geographic distribution	Orthohantavirus	Association with human disease	Additional details	References
*Akodon azarae*	Uruguay, Eastern Argentina, Paraguay, and southern Brazil.	*O. andesense* (Pergamino genotype)	Unknown	Inhabits open and wet grasslands along rivers and marshes, open thorn woodlands, and borders of cultivated fields. Present in crops and pastures.	Busch and colleagues (2019) [28] Gómez Villafañe and colleagues (2022) [29] ICTV (2022) [30] Levis and colleagues (1998) [31] Maroli and colleagues (2015) [32] Maroli and colleagues (2018) [33] Pardiñas and colleagues (2015) [34] Vadell and colleagues (2011) [35]
*Akodon montensis*	Northeastern Argentina, Paraguay, Brazil.	Jaborá (Paraguayan genotype Apé Aimé synonymized under Jaborá by Rivera and colleagues, 2015).	Unknown	Present in gallery forests along rivers or wetlands. It occupies diverse areas, from primary forests to highly disturbed ones.	Bellomo and colleagues (2024) [36] Burgos and colleagues (2021) [37] Carvalho de Oliveira and colleagues (2011) [38] Pardiñas and colleagues (2003) [39] Vadell and colleagues (2024) [40] Rivera and colleagues (2015) [7]
*Calomys callosus*	Argentina, Paraguay, eastern Bolivia, central-western Brazil	*O. negraense*	HPS	Present in the Chaco ecoregion and transitions with Brazilian Cerrado and Bolivian Dry Forest.	Almeida and colleagues (2007) [41] Carrol and colleagues (2005) [42] González-Ittig and colleagues (2022) [43] Salazar-Bravo (2015) [44]
*Calomys fecundus*	Northwestern Argentina, southern Bolivia	*O. negraense*	HPS in Argentina	Found in the ecotone between Southern Andean Yungas and Dry Chaco. Also present in crops.	Levis and colleagues (2004) [45] Pini and colleagues (2012) [46] Pinotti and colleagues (2020) [47]
*Calomys laucha*	Northern Paraguay, southeastern Bolivia, central Argentina, and Brazil	*O. negraense*	HPS in Paraguay	Inhabits grasslands, crops, forest fringes, and has high tolerance to disturbed habitats. Has also been described in southern Brazil and Uruguay, but these populations may represent a different cryptic species.	González-Ittig and colleagues (2014) [48] Johnson and colleagues (1997) [49] Teta and colleagues (2017) [27] Travassos da Rosa and colleagues (2012) [50] Yahnke and colleagues (2001) [51]
*Holochilus chacarius*	Central-eastern and north-eastern Argentina, western Paraguay, and Brazil.	Alto Paraguay	HPS	Registered in lagoons, flooded grasslands, esparto grasslands, and reedbeds of the Humid Chaco. The subspecies *H. chacarius balnearium* is found in northwestern Argentina, and *H. chacarius chacarius* has a wider eastern distribution and is the reservoir host of the virus.	Bellomo and colleagues (2021) [52] Fortabat (2001) [53] Gonçalves and colleagues (2015) [54] Martin and colleagues (2024) [55] Massa and colleagues (2019) [56] Prado and colleagues (2021) [57] Rimoldi and Cimento (2021) [58]
*Necromys lasiurus*	Northern Argentina, western and central-western Paraguay, central and central-eastern Brazil.	*O. andesense* (Maciel and Araraquara genotypes)	Araraquara causes HPS in Brazil Maciel virus not linked to human cases.	Currently synonymized with *N. benefactus*. Found mainly in open areas such as savannahs, grasslands, and dry forests. Adapted to disturbed areas.	Enria and colleagues (2004) [59] Figueiredo and colleagues (2014) [60] Pardiñas and colleagues (2015) [61]
*Oligoryzomys chacoensis*	Southeastern Bolivia, western Paraguay, southwestern Brazil, and northern Argentina.	*O. andesense* (Orán genotype)	HPS	Present in the Chaco ecoregion and semi-evergreen dry forests.	Levis and colleagues (1998) [31] González-Ittig and colleagues (2014) [62]
*Oligoryzomys flavescens sensu stricto*	Central and northeastern Argentina and southwestern Uruguay.	HU39694, *O. andesense* (Lechiguanas, Buenos Aires and Central Plata genotypes)	HPS	Part of the *Ol. flavescens* species complex. Prefers areas close to water streams and humid lowlands with dense and high weeds. Tolerant to disturbed habitats	Bonaventura and colleagues (2003) [63] Gómez Villafañe and colleagues (2024) [64] Levis and colleagues (1998) [31] Maroli and colleagues (2022) [65] Martinez and colleagues (2020) [66] Rivera and colleagues (2018) [67] Vadell and colleagues (2011) [35] Vadell and colleagues (2024) [40]
*Oligoryzomys longicaudatus*	Southern Argentina and central-southern Chile	*O. andesense* (Andes genotype)	HPS	Can transmit person-to-person. Diverse habitats such as temperate and sub-Antarctic forests, scrubs, grasslands, steppe and river margins, streams, and wetlands. Also present in disturbed areas.	Andreo and colleagues (2012) [68] González-Ittig and colleagues (2010) [69] Jayat and colleagues (2011) [70] Padula and colleagues (2002) [71] Polop and colleagues (2010) [72]
*Oligoryzomys nigripes*	Central and southeastern Brazil, eastern Paraguay and Uruguay, and northeastern and eastern Argentina	Juquitiba, *O. andesense* (Lechiguanas genotype)	HPS	Inhabits primary and secondary vegetation in the Atlantic Forest.	Padula and colleagues (2007) [73] Delfraro and colleagues (2008) [74] Vadell and colleagues (2011) [35] Colombo and colleagues (2019) [75] Martinez and colleagues (2005) [76] Rivera and colleagues (2015) [7]
*Oligoryzomys occidentalis* *(Ol. flavescens occidentalis*)	Central-western and northwestern Argentina, southern and central-western Bolivia	*O. andesense* (Bermejo genotype)	HPS	Part of the *Ol. flavescens* species complex. It is a rare to moderately frequent species in its distribution range. In the Dry Chaco, it is found in greater density in humid places and associated with water bodies.	González-Ittig and colleagues (2010) [69] González-Ittig and colleagues (2014) [62] Jayat and colleagues (2011) [70] Padula and colleagues (2002) [71]
*Scapteromys aquaticus*	Central-east and northern Argentina and southern Paraguay.	Leyes	Unknown	Inhabits wetlands and flooded lands, present in disturbed habitats.	Bellomo and colleagues (2021) [52] de Oliveira and colleagues (2015) [77]
*Rattus norvergicus*	Worldwide (especially present in Europe, Asia, Americas)	Seoul virus (SEOV)	HFRS (in Asia and Europe)	Often associated with urban areas	Fitte and colleagues (2023) [78]Seijo and colleagues (2023) [79]

### Spillover hosts and multi-host infections

Besides the 14 reservoir hosts found for SSA, several rodent species were either documented as seropositive or viral RNA was detected in them. However, since they did not meet the criteria to be a reservoir host, they were considered as spillover hosts. It should be noted that several of these species may become reservoir hosts once sufficient evidence has been accumulated. Spillover events refer to the incidental transmission of a pathogen from its primary (reservoir) host to other species that do not maintain the infection over time. Such events are more likely to occur when rodent hosts share sympatric ranges, exhibit similar ecological niches, or are phylogenetically related, facilitating horizontal transmission through direct or indirect contact [2]. Spillover events seem to be more common for cricetid-borne orthohantaviruses than other orthohantavirus-host groups, generating frequent opportunities for host-switching events [81]. Alternative host species may play a significant role in viral transmission dynamics through limited or even long-term outbreaks, and further investigation of the importance of alternative hosts in orthohantavirus ecology is necessary [81].

In Table 2, spillover hosts are grouped according to shared orthohantavirus genotypes and considering the ecological and phylogenetic contexts (such as sympatric ranges and potential for horizontal transmission). This approach reflects the role of seropositive rodents as part of a multi-host system, which may include both maintenance and incidental hosts. Determining whether a species is a true reservoir requires longitudinal studies that confirm sustained viral replication and transmission within the host population [14–16].

**Table 2:** Sporadic hosts documented in the bibliography grouped by orthohantavirus genotype.

Sporadic hosts	Spillover region	Orthohantavirus	Reservoir host	Additional details	References
*Akodon simulator* *Oligoryzomys chacoensis* *Euryoryzomys legatus*	Northwestern Argentina	*Orthohantavirus negraense*	*Calomys fecundus*	*A. simulator* is distributed along the lower Andean slopes of southern Bolivia and northwestern Argentina and may represent a species complex that includes the taxa previously named *Ak. simulator*, *Ak. glaucinus*, and *Ak. tartareus*. *E. legatus* is distributed in premontane and montane forests along the eastern Andean slope in extreme southern Bolivia and northernmost Argentina.	Cassinelli and colleagues (2024) [82] Jayat and Ortiz (2019) [83] Levis and colleagues (2004) [45] Percequillo (2006) [84] Pini and colleagues (2012) [46]
*Abrothrix longipilis* *A. hirta* *A. olivacea* *Loxodontomys microtus* *Rattus rattus* *Rattus norvegicus*	Andes Mountains in central and southern Chile and Argentina	*Orthohantavirus andesense* (Andes genotype)	*Oligoryzomys longicaudatus*	*A. longipilis* ranges from central chile and central-western Argentina to the extreme south of SA. Abundant in dense *Nothofagus* forests, also occurs in most other habitats in the region. *A. hirta* shows a wide geographic distribution in Chile and Argentina—from 35° S to 55° S—and occupies diverse habitats, including xerophilous forests, pre-Andean shrublands, temperate rainforests, and arid Patagonian steppe. *A. olivacea* extends over central and southern Chile and Argentina, inhabiting grasslands, shrublands, forests, rocky areas, and mountainous sites. *Loxodontomys micropus* is distributed in southern Chile and southwestern Argentina at elevations up to 2500 m. *Rattus rattus and Rattus norvegicus* are highly invasive rodents with an almost worldwide distribution.	Andreo and colleagues (2012) [68] Cantoni and colleagues (2001) [85] Doherty and colleagues (2022) [86] Fernández and colleagues (2008) [87] Patterson and colleagues (2015) [88] Teta and colleagues (2017) [89] Valdez and Délia (2021) [90]
*Oxymycterus rufus*	Paraná Flooded Savanna	*Orthohantavirus andesense* (Pergamino genotype)	*A. azarae*	No disease has been formally linked to it. *Ox. rufus* is native to north-eastern and eastern Argentina, Paraguay, Brazil, and Uruguay. It inhabits the Paraná River Delta, coastal areas, and extends inland in eastern Argentina.	Colombo and colleagues (2019) [75] Gómez-Villafañe and colleagues (2019) [91] Gómez-Villafañe and colleagues (2022) [29] ICTV (2022) [30] Levis and colleagues (1998) [31] Maroli and colleagues (2023) [92]
*Oxymycterus quaestor* *Oxymycterus nasutus* *Thaptomys nigrita* *Ak. paranaensis*	Southern Brazil and Uruguay	*Orthohantavirus andesense* (Juquitiba genotype)	*Oligoryzomys nigripes*	*Oxymycterus quaestor* is distributed in southeastern Paraguay, northeastern Argentina, and southeastern Brazil. The forms *Ox. judex* and *Ox. quaestor* were synonymized under the name *Ox. quaestor*. Inhabits primary forests but has also been documented in forests with disturbed vegetation. *Ox. nasutus* inhabits grasslands of Uruguay and south-eastern Brazil. *Thaptomys nigrita* is found in southeastern Brazil and Paraguay, and northwestern Argentina. It has strong fossorial tendencies—spending much of its time burrowing or foraging underground—which may reduce direct contact with the reservoir host.	Carvalho de Oliveira and colleagues (2014) [10] Delfraro and colleagues (2008) [74] Galliari and Pardiñas (2021) [93] Padula and colleagues (2007) [73] Peçanha and colleagues (2019) [94] Raboni and colleagues (2012) [95] Rivera and colleagues (2015) [7] Teta and colleagues (2015) [96]
*Ratus rattus*	Worldwide	*Orthohantavirus seoulense*	*R. norvergicus*	Found in tropical and subtropical regions	Fitte and colleagues (2023) [78]
*Bibimys chacoensis* *Deltamys kempi* *Graomys chacoensis* *Akodon toba*	Various	Undetermined		*Bibimys chacoensis* is endemic to gallery forests and open areas of the Humid Chaco ecoregion. *Graomys chacoensis* (previously misidentified as *G. griseoflavus)* inhabits semi-arid to humid shrub and forest areas across the Chaco region of western Paraguay, southeastern Bolivia, and central and northern Argentina.	Chu and colleagues (2003) [97] D’Elía and Pardiñas (2016) [98] González and Martinez-Lanfranco (2011) [99] Gómez-Villafañe and colleagues (2022) [29] Musser and Carleton (2005) [100] Pardiñas and Teta (2015) [101] Puerta and colleagues (2006) [102] Williams and colleagues (1997) [103]

### Genotype diversity in Southern South America

The evolution of the genus *Orthohantavirus* in SSA is characterized by a great genetic diversity and a wide geographic distribution, with two main phylogenetic clades (Andes and Andes-like; and Laguna Negra and Laguna Negra-like viruses) influenced by host switching events and the geographic proximity of rodent hosts [7,17]. Phylogenetic evidence suggests that the ancestor of orthohantaviruses, which are hosted by sigmodontine rodents, originated in Brazil approximately 400,000 years ago. Subsequently, it spread westward (to Bolivia, Peru, and Paraguay), where it gave rise to the Laguna Negra clade, and southward (to Argentina and Chile), leading to the emergence of the Andes clade [104].

While our revision allowed us to identify 15 distinct viral genotypes hosted by 14 reservoir species in SSA, only three orthohantavirus species are officially recognized by the ICTV: *Orthohantavirus negraense*, *O. andesense*, and *O. seoulense*. Many of the viral genotypes detected were classified under *O. andesense* by the ICTV (Andes *sensu stricto*, Bermejo, Lechiguanas, Maciel, Orán and Pergamino), while others (Araraquara, Buenos Aires, Juquitiba and HU39694) were associated with this clade in phylogenetic studies [105–107]. Additionally, the Jaborá and Alto Paraguay genotypes are not grouped under any officially recognized viral species and display significant internal genetic diversity [55,106,108].

**Fig 2:**
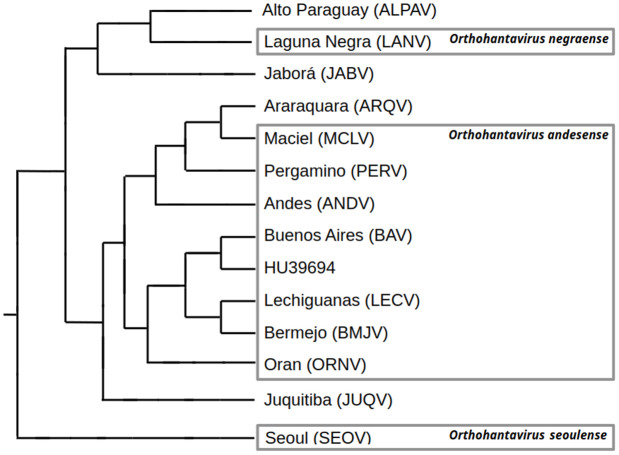
Illustrative tree of the phylogenetic relationships described for orthohantavirus genotypes present in Southern South America. The topology is based on a tree containing whole genomes [107] with the addition of Alto Paraguay and Araraquara virus according to previous studies [55,106].

To facilitate the description of the found genotypes, the current knowledge of their phylogeny is represented in Fig 2. Since the ICTV Hantaviridae Study Group decided to reassess the entire Hantaviridae family using stringent criteria, that implies assessing only viruses for which their complete or near-complete genome is available [109], we used a tree containing the whole-genome as a base topology [107]. Besides, according to phylogenies of the S-segment [55,106,108], we defined the phylogenetic position of the Alto Paraguay and Araraquara genotypes in the illustrative tree. We did not include the Leyes genotype in this tree because its phylogenetic position is still uncertain [52].

One of the recognized species, *Orthohantavirus negraense* (LANV, originally called Laguna Negra [30]), was first isolated in rodents of the species *C. laucha* from Paraguay. This virus shows higher incidence of human infection and lower clinical severity than *O. andesense* [49,103]. No genotypes have been described within this viral species, although it has several reservoirs such as *C. fecundus* in Argentina, *C. callosus* in Bolivia and the mentioned *C. laucha* in Paraguay (Table 1) [42,45,46,48,49].

Regarding the Andes and Andes-like orthohantavirus*,* to comply with the ICTV-mandated binomial format, the original name was replaced with *Orthohantavirus andesense* [30]. In SSA, six genotypes have been recognized within this viral species: Andes (ANDV), Bermejo (BMJV), Lechiguanas (LECV), Maciel (MACV), Orán (ORNV), and Pergamino (PERV) (Fig 2) [110]. The Andes genotype (ANDV) *sensu stricto* is considered one of the most lethal orthohantaviruses in Argentina, with a case fatality rate exceeding 36% and intensive care required in 96% of the reported cases. It has also been proposed as capable of person-to-person transmission, although this remains a subject of debate [66,76,111–113]. The Lechiguanas clade within this species includes the genotypes Bermejo, Ñeembucú, Lechiguanas *sensu stricto*, and Central Plata. The Bermejo and Ñeembucú genotypes are found in northwestern Argentina and western Paraguay, while Lechiguanas and Central Plata were identified in Argentina and Uruguay [7]. While multiple studies have demonstrated person-to-person transmission for the Andes genotype, evidence supporting this mode of transmission for the Lechiguanas genotype remains limited and inconclusive [76]. Although full genome sequencing is still lacking for several genotypes that are presumed to belong to the *O. andesense* clade, it is likely that they will be formally classified within this group once complete genome data become available. This is the case for the Araraquara, Buenos Aires, HU39694, and Juquitiba genotypes [114]. The Araraquara genotype appears to be one of the most virulent orthohantaviruses in Brazil, with a mortality rate of 50% [99]. The genotype HU39694 was initially described as an independent lineage, related to the Lechiguanas and Bermejo genotypes [100]. However, it was later included within the Buenos Aires genotype (BAV). Both HU39694 and BAV belong to the *O. andesense* clade [107]. The HU39694 genotype was first characterized from a human case in Buenos Aires Province [115], and was later associated with the rodent *Oligoryzomys flavescens* [100]. BAV is of great concern due to its implication in several outbreaks and suspicion of person-to-person transmission [107]. It is interesting to note that human cases from Tucumán province (north-western Argentina) associated with HU39694 genotype were reported, but the rodent reservoir could not be identified since specific orthohantavirus antibodies were not detected in the captured individuals [116]. The Juquitiba clade (JUQV) includes strains with the names Juquitiba or Araucaria. The Juquitiba genotype was originally detected in a patient with HPS [117]. Later on, it was found in other humans and in specimens of *Ol. nigripes* and *Ox. nasutus* [73]. About 85% of the HPS cases reported in Brazil have occurred in a region where *Ol. nigripes*, the reservoir of JUQV, is frequent. Phylogeographic studies revealed that JUQV includes two major clades, one with sequences from Rio de Janeiro, Espírito Santo and Goiás states and other with a widespread geographic distribution in southern Brazil, Argentina, and Paraguay [106]. The latter country has two monophyletic strains, the Canindeyú and Itapúa, named according to the location they were first detected [108].

The viral species *Orthohantavirus seoulense* is a known causative agent of HFRS in the Old World [2]. However, no clinical cases of HFRS have been reported in SSA to date, although seropositive rodents for *O. seoulense* were detected in eco-epidemiological studies, particularly *Rattus rattus* and *R. norvegicus* [78,79].

Although several described genotypes have not yet been officially recognized as species, it has been shown they are lineages forming monophyletic clades. This is the case of Alto Paraguay (ALPAV) and Jaborá (JABV) (Fig 2). The Alto Paraguay genotype was originally detected in rodents identified as *H. chacarius chacarius* in Paraguay [97]. Later, both the circulation of ALPAV in the same rodent species and a human case were demonstrated in Santa Fe province, Argentina [55]. The Jaborá genotype has not been associated with human cases [118]. The genotypes Apé Aimé, Itapua, and AC210py were synonymized under the name Jaborá [7]. The phylogeographic analysis of JABV revealed two clades, one composed of sequences from rodents from Brazil, and another one of sequences retrieved from Paraguay [106].

Finally, in a recent work [52], individuals of the rodent *Scapteromys aquaticus* were found infected with a novel virus. Preliminary genetic characterization analysis could not match this new virus to any other known orthohantavirus. Therefore, the authors proposed to name it as Leyes virus because all positive rodents were captured over the margins of the Leyes stream (Santa Fe province, Argentina). Furthermore, a potentially new pathogenic orthohantavirus strain has recently been described from a human case in the province of Tucumán, Argentina. The virus showed low nucleotide similarity to known orthohantaviruses, suggesting a novel genotype. Phylogenetic analysis revealed that this strain clustered with sequences previously obtained from *Oligoryzomys* rodents captured in north-western Argentina, which had not been published [119].

### Ecological and environmental drivers of Orthohantavirus transmission

Orthohantaviruses represent a significant public health concern due to their zoonotic transmission from wild rodent hosts to humans. In SA, the rodent species involved in the orthohantavirus-human dynamic are typically generalist, highly abundant, and well adapted to anthropogenically disturbed environments [92,120]. Understanding the ecological, environmental, and social factors that influence virus maintenance and transmission is key to informing prevention and control strategies, particularly in regions like SSA, where surveillance and diagnostic programs are still developing and mostly constrained by resource availability.

Agriculture, deforestation, mining, and urban expansion have profoundly transformed natural habitats in SSA, often creating conditions that favor the proliferation of reservoir species [60,121,122]. These rodent hosts are often habitat generalists that thrive in fragmented or altered landscapes. This leads to increased local population densities and more frequent interactions between humans and rodents [51,123], subsequently enhancing opportunities for viral transmission. For example, it has been shown that habitat alteration through human land use can lead to expanded or restructured distributions of both rodent reservoirs and orthohantaviruses, enhancing circulation in rural and fragmented areas [122]. In the Paraná Delta (Argentina), a region that has undergone significant land use changes, it was found that rodent population density rose due to such habitat modifications, increasing the likelihood of orthohantavirus transmission [124].

Host characteristics, such as preferred diet can influence exposure, as some food sources may be more easily contaminated with virus particles. For example, *Ol. longicaudatus* abundance has been positively associated with exotic plant species like *Rosa rubiginosa*, which serves both as shelter and food for the rodent [68,123]. Demographic traits such as age and sex may determine orthohantavirus prevalence. Older rodents and males tend to have higher infection rates, likely due to increased territoriality and aggression during breeding seasons, which facilitate horizontal transmission via wounding and contact [17,125]. Aggressive encounters appear to be important for intrasexual transmission, and social grooming or copulation provide opportunities for intersexual transmission [81].

Beyond ecological conditions, socio-economic and demographic contexts shape the risk of human infection. Factors such as housing quality, hygiene practices, and occupational exposure can increase human vulnerability [12]. Sociodemographic studies have identified ethnicity, occupation, and sex as key risk factors for hantavirus infection outcomes in SA. In Chile, individuals of European descent were found to have a 5.1-fold higher risk of developing severe disease compared to those of Amerindian ancestry [126]. A similar pattern was observed in Paraguay and Argentina, where seroprevalence of orthohantavirus antibodies was higher among indigenous populations, indicating elevated exposure rates; despite most clinical cases occurred in non-indigenous individuals [103,127,128]. Rural residency and agricultural work have also been associated with increased risk due to higher exposure to infected rodents [8]. Moreover, a higher incidence of disease has been consistently reported among men compared to women [129,130].

Climatic factors are also closely linked to rodent population dynamics and orthohantavirus transmission. Increased precipitation fosters vegetation growth and food availability, which in turn promotes rodent population growth. Temperature influences reproduction rates, survival, and the persistence of the virus in the environment; lower temperatures prolong viral viability on surfaces and excreta [120,131,132]. In Argentina, the abundance of *Ol. flavescens* was associated with temperature, rainfall, and the proximity to freshwater bodies [133], while the seasonal dynamics of *Akodon azarae* were influenced by vegetation cover and rainfall [134]. Large-scale climatic events like the El Niño Southern Oscillation or the Antarctic Oscillation can cause vegetation pulses that facilitate rodent outbreaks, which have been associated with spikes in orthohantavirus prevalence and subsequent human infections [20,60].

### Transmission routes and prevention strategies

Orthohantaviruses are primarily transmitted to humans via inhalation of aerosolized viral particles from rodent excreta (urine, saliva, and feces), as well as through direct contact with contaminated surfaces or bites [2,135]. Transmission of cricetid-borne orthohantaviruses is thought to occur predominantly via saliva, with a comparatively minor contribution from urine and feces [81]. Within rodent populations, both horizontal and vertical transmission occur, with horizontal (*e.g*., through aggressive interactions) being particularly important in sustaining the virus [7,81]. While most human infections result from rodent exposure, person-to-person transmission has been documented for the Andes virus lineage, although such cases remain rare and generally involve close contact [98,111–113]. Prevention efforts in SA have focused on early outbreak detection, public education, and reducing human-rodent contact. Simple and low-cost interventions are recommended: use of personal protective equipment in high-risk settings, rodent-proofing dwellings, and safe cleanup of rodent excreta [12]. Community-based education is also essential, including hygiene promotion, rodent avoidance practices, and maintaining clean peridomestic environments [136]. Ultimately, integrating ecological and epidemiological research through modeling and long-term field studies is fundamental for predicting outbreaks and developing sustainable control strategies [2].

## Conclusion

This work highlights the diversity of the *Orthohantavirus* genus and their hosts in SSA, identifying 15 viral genotypes hosted by 14 reservoir rodent species. In fact, although **O. andesense*, *O. negraense*,* and *O. seoulense* are the only three viral species officially recognized, several other genotypes have been identified and may eventually be considered for formal taxonomic classification. Regarding the previous review conducted in SSA [21], our research provides several important updates, underscoring the value of revisiting and consolidating current knowledge. For instance, a new viral genotype (Leyes virus) was discovered in *Scapteromys aquaticus*, and *C. fecundus* was identified as a reservoir of Laguna Negra virus. Moreover, many genotypes formerly considered unclassified have been included within the *O. andesense* species. Additionally, some rodent species previously proposed as reservoir hosts are now considered spillover hosts, as in the case of *Ox. nasutus* for the Juquitiba genotype. It should be noted that the viruses and hosts mentioned here are not a definitive list, since new findings about the distribution and taxonomy of both viral strains and their hosts constantly emerge. New reservoir species and viral genotypes will likely be discovered [137], and the ranges of viruses and hosts may also shift over time due to factors like climate or land use and land cover changes [8]. For example, the range of the orthohantavirus Lechiguanas, hosted by *Ol. flavescens* and **Ol. nigripes*,* has recently expanded to Corrientes province in north-eastern Argentina [64]. Also, the epidemiological knowledge of viral strains is constantly being updated. For example, the ALPAV, previously considered nonpathogenic, was reported for the first time as pathogenic in Argentina [52]. This implies an expansion of the known HPS endemic area in central Argentina and a greater diversity of orthohantavirus in the country than previously reported.

The diversity of orthohantavirus genotypes and their rodent hosts presents a challenging epidemiological scenario. The Andes genotype, linked to *Ol. longicaudatus*, is particularly concerning due to its suspected ability to be transmitted between humans and its high mortality rate. Other genotypes, such as Lechiguanas and HU39694, are also major contributors to HPS cases in the region. The geographic distribution of these viruses is closely tied to the ecology of their rodent hosts, many of which inhabit a wide range of environments, including human-modified landscapes. For example, species of the genera *Akodon*, *Calomys*, and *Oligoryzomys* are present in anthropized habitats, increasing the risk of human exposure to these pathogens. Our synthesis highlights the critical need for ongoing surveillance and research to better understand the public health risks and guide space-specific and timely prevention strategies and interventions.
